# Empathic and Self-Regulatory Processes Governing Doping Behavior

**DOI:** 10.3389/fpsyg.2017.01495

**Published:** 2017-09-22

**Authors:** Ian D. Boardley, Alan L. Smith, John P. Mills, Jonathan Grix, Ceri Wynne

**Affiliations:** ^1^School of Sport, Exercise and Rehabilitation Sciences, University of Birmingham Birmingham, United Kingdom; ^2^Department of Kinesiology, Michigan State University, East Lansing MI, United States; ^3^University of Chichester Chichester, United Kingdom

**Keywords:** performance enhancing drugs, moral disengagement, self-regulatory efficacy, empathy, mediation, multisample analyses, sport, exercise

## Abstract

Evidence associating doping behavior with moral disengagement (MD) has accumulated over recent years. However, to date, research examining links between MD and doping has not considered key theoretically grounded influences and outcomes of MD. As such, there is a need for quantitative research in relevant populations that purposefully examines the explanatory pathways through which MD is thought to operate. Toward this end, the current study examined a conceptually grounded model of doping behavior that incorporated empathy, doping self-regulatory efficacy (SRE), doping MD, anticipated guilt and self-reported doping/doping susceptibility. Participants were specifically recruited to represent four key physical-activity contexts and consisted of team- (*n* = 195) and individual- (*n* = 169) sport athletes and hardcore- (*n* = 125) and corporate- (*n* = 121) gym exercisers representing both genders (*n_male_* = 371; *n_female_* = 239); self-reported lifetime prevalence of doping across the sample was 13.6%. Each participant completed questionnaires assessing the aforementioned variables. Structural equation modeling indicated strong support for all study hypotheses. Specifically, we established: (a) empathy and doping SRE negatively predicted reported doping; (b) the predictive effects of empathy and doping SRE on reported doping were mediated by doping MD and anticipated guilt; (c) doping MD positively predicted reported doping; (d) the predictive effects of doping MD on reported doping were partially mediated by anticipated guilt. Substituting self-reported doping for doping susceptibility, multisample analyses then demonstrated these predictive effects were largely invariant between males and females and across the four physical-activity contexts represented. These findings extend current knowledge on a number of levels, and in doing so aid our understanding of key psychosocial processes that may govern doping behavior across key physical-activity contexts.

## Introduction

Understanding the psychosocial processes that may explain the use of prohibited performance enhancing substances or methods – often referred to as doping – is important in both sport and exercise contexts. In sport, doping represents an unfair performance advantage over competitors because it is against the rules. Further, exercisers in gymnasia who use performance enhancing drugs (PED) are at increased risk of the numerous adverse health consequences associated with their use (Pope et al., 2014). Although accurate prevalence rates are difficult to determine, a recent article summarizing the best available evidence estimated prevalence of doping in adult elite sport to be between 14 and 39% (de Hon et al., 2015)^1^. An important aim for researchers investigating doping is to identify and understand psychosocial factors that influence the likelihood of athletes and exercisers using illicit performance enhancing substances. The current research sought to contribute to the literature on this topic by testing a conceptually grounded process model of doping behavior^2^ underpinned by the social cognitive theory of moral thought and action (Bandura, 1991).

According to Bandura (1991), transgressive activities – such as doping – are deterred when people anticipate experiencing negative emotional reactions (e.g., guilt) as a result of engaging in them. As doping is against the rules of sport (World Anti-Doping Agency [WADA], 2015), and often viewed as morally wrong by exercisers (Probert and Leberman, 2009), sport and exercise participants may anticipate feeling guilty if they decide to dope. Anticipation of such unpleasant emotional reactions should therefore deter them from engaging in doping. However, Bandura (1991) also explained how people can reduce or eliminate anticipation of such negative emotional reactions through use of any of eight psychosocial mechanisms; use of these mechanisms is collectively referred to as moral disengagement (MD). Representing the conditional endorsement of transgressive acts, MD may facilitate doping by allowing athletes and exercisers to use prohibited substances or methods without experiencing negative emotional reactions such as guilt.

Importantly, research evidence associating doping with MD has emerged over the previous decade. For instance, a series of qualitative studies have provided evidence of MD in exercisers and athletes who had doped. First, Boardley and Grix (2014) conducted semi-structured interviews with nine PED-using bodybuilders. Interviews centered on psychosocial processes facilitating doping, and deductive content analysis revealed evidence of six of the eight MD mechanisms (i.e., moral justification, euphemistic labeling, advantageous comparison, displacement of responsibility, diffusion of responsibility and distortion of consequences).^3^ One weakness of this study was that participants all originated from a single gym. Thus, Boardley et al. (2014) then conducted a follow-up study with 64 male bodybuilders with experience of doping from across England. Consistent with the initial study, deductive content analysis of the study data revealed evidence of the same six MD mechanisms. These findings were then extended to sport by Boardley et al. (2015), who interviewed twelve male athletes with experience of doping from a variety of team- and individual-sports. Researchers have also identified positive links between MD, intention to dope, and reported doping across a small number of quantitative studies (e.g., Lucidi et al., 2004, 2008; Zelli et al., 2010). However, it is important to note these studies were all conducted with high-school students, a significant proportion (43.0 – 45.2%) of whom did not partake in any extracurricular sport. As such, the relationship between doping and MD has not been statistically examined with participants from key sport and exercise contexts, including those in which prevalence rates for doping are likely to be much higher. Further, key variables (e.g., anticipated guilt) from Bandura’s (1991) theory were not examined in these studies. Thus, currently there is an absence of research that specifically targets participants from key sport and exercise contexts, and examines the effects of MD on doping within models that consider key theoretically grounded influences and outcomes of MD.

To better capture the processes that may explain how doping MD may influence doping behavior, research is needed that investigates potentially influential empathic and self-regulatory processes. One such process purported by Bandura (1991), suggests MD impacts upon transgressive behaviors through its effect on regulatory emotions such as guilt. Guilt represents a distasteful emotional state experienced as tension and regret resulting from the personal responsibility felt – and empathic feelings for – someone suffering anguish (Hoffman, 2000). Due to its unpleasant connotations, guilt can be adaptive in regulating harmful conduct, as people are deterred from engaging in behaviors they anticipate will result in guilt (Bandura, 1991). Support for the adaptive role of guilt is evidenced by negative relationships between proneness to experience guilt and aggression (Stuewig et al., 2010) and bullying behavior (Mazzone et al., 2016). Importantly, anticipation of guilt is thought to be diminished by MD, which involves portraying transgressions favorably, reducing personal accountability, and downplaying their detrimental consequences (Bandura, 1991). Consistent with this, work in and out of sport (Bandura et al., 1996; Stanger et al., 2012) supports the notion that anticipation of guilt may be reduced by MD. Therefore, athletes and exercisers with higher levels of doping MD may have lower levels of anticipated guilt for PED use, which in turn may be linked with an increased likelihood to adopt doping practices. Thus, any positive effect of MD on doping may be mediated – at least in part – by reductions in anticipated guilt.

As well as potential outcomes, it is also important to investigate potential antecedents of MD, as such research may inform interventions seeking to reduce it. One potential antecedent of MD is empathy, which represents a tendency to vicariously experience emotional and cognitive responses to another individual’s emotional state (Davis, 1983, 1994). A lack of empathy implies an inability to view the world from another individual’s perspective or to feel sympathy toward them (Davis, 1994). Empathy is thought to impair MD because endorsement of deleterious conduct is more difficult when one can anticipate and experience the consequences of one’s detrimental actions toward others (Feshbach, 1975; Bandura, 1986, 1991; Hoffman, 2000; Paciello et al., 2013). Increased empathy may therefore be linked with a reduced likelihood of transgressive and harmful behavior through a negative influence on MD. Consistent with these propositions, negative relationships between empathy and transgressive conduct in sport have been demonstrated (Kavussanu et al., 2009; Stanger et al., 2012), and research out of sport has negatively linked empathy and MD (Paciello et al., 2013). Thus, higher levels of empathy in athletes and exercisers should be associated with lower levels of MD, which in turn ought to be linked with reduced doping.

Another variable that may influence athletes’ and exercisers’ MD is self-regulatory efficacy (SRE), which represents one’s ability to resist personal and social pressures to engage in detrimental conduct (Bandura et al., 2001). Importantly, Bandura et al. (2001) proposed increased SRE should lead to lower levels of MD, because those who have strong beliefs in their ability to resist incentives to engage in harmful conduct have no need to justify and rationalize such behavior. When specifically applied to doping, SRE represents one’s capacity to withstand the personal and social influences that encourage PED use. In accord with Bandura’s theorizing, research by Lucidi and colleagues – introduced earlier – has shown that doping SRE negatively predicts intention to dope in Italian adolescents (Lucidi et al., 2008; Zelli et al., 2010). However, as mentioned earlier the findings of these studies are limited due to their use of high-school students, many of whom (43–45.2%) were not partaking in any extracurricular sport. Additionally, Lucidi and colleagues considered the predictive abilities of MD alongside SRE rather than as a mediator of the effects of SRE on doping, which is inconsistent with the causal pathway hypothesized and empirically supported by Bandura et al. (2001). As such, research testing relationships accurately grounded in theory and specifically sampling participants from key sport and exercise contexts is needed.

**FIGURE 1:**
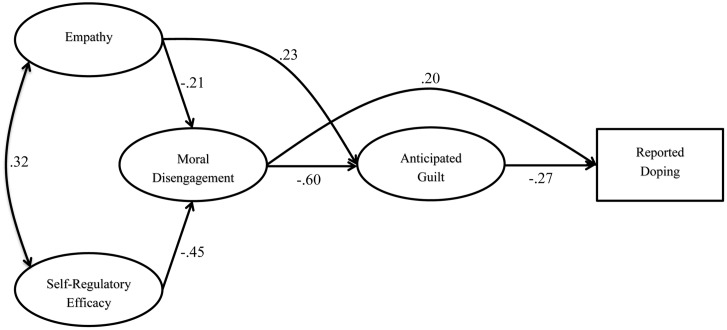
Structural model including parameter estimates (*N* = 610). For all parameter estimates, *p* < 0.05.

The primary aim of the current research was to test a model of doping behavior grounded in Bandura’s (1991) theory with team- and individual-sport athletes and corporate- and hardcore-gym exercisers. Based on the arguments presented to this point, the model (see **Figure 1**) proposed empathy and doping SRE would negatively predict doping MD, that doping MD would negatively predict anticipated guilt, and that anticipated guilt would negatively predict reported doping (Bandura, 1986, 1991; Bandura et al., 1996, 2001; Lucidi et al., 2008; Zelli et al., 2010; Stanger et al., 2012; Paciello et al., 2013). Further, we anticipated that doping SRE and empathy would negatively predict doping indirectly via changes in doping MD and anticipated guilt. In addition to its indirect effect, we also expected empathy to have a direct positive predictive effect on anticipated guilt. Finally, doping MD was expected to positively predict doping both directly (i.e., to account for any predictive effects of MD on doping that operate through emotions other than guilt [e.g., shame]) and through a mediated effect via anticipated guilt (Bandura, 1991; Bandura et al., 1996). A secondary aim of the research was to test the structural invariance of the proposed model between males and females and across the four sport/exercise groups represented. However, as low levels of doping behavior were anticipated in some sub-groups (e.g., females, corporate-gym exercisers), doping susceptibility replaced reported doping for the multisample analyses addressing this research aim.

## Materials and Methods

### Participants^4^

To follow are seven sample descriptions. The first is for the entire sample, whereas the subsequent six are for sub-divisions of the full sample employed during the multisample analyses.

#### Full Sample

Participants were team- (e.g., American football, soccer, field hockey; *n* = 195) or individual- (e.g., athletics, swimming, triathlon; *n* = 169) sport athletes or hardcore- (*n* = 125) or corporate- (*n* = 121) gym exercisers, representing both genders (*n_male_* = 371; *n_female_* = 239), with ages ranging from 16 to 73 years (*M* = 26.27, *SD* = 10.84). They had been training/competing for an average of 8.10 years (*SD* = 7.12), spent an average of 8.29 h (*SD* = 4.48) per week training, and had trained in their current gym/with their current team for an average of 3.97 years (*SD* = 4.73). 527 (86.4%) participants reported never having used PEDs, 46 (7.5%) had used them prior to the past 3 months, 20 (3.3%) had used them in the past 3 months and 17 (2.8%) were current users.

#### Male Participants

Participants were team- (*n* = 135) or individual- (*n* = 88) sport athletes or hardcore- (*n* = 102) or corporate- (*n* = 46) gym exercisers, with ages ranging from 16 to 73 years (*M* = 25.91, *SD* = 10.49). They had been training/competing for an average of 7.81 years (*SD* = 7.56), spent an average of 8.68 h (*SD* = 4.53) per week training, and had trained in their current gym/with their current team for an average of 3.90 years (*SD* = 4.65). 304 (81.9%) participants reported never having used PED, 36 (9.7%) had used them prior to the past 3 months, 17 (4.6%) had used them in the past 3 months, and 14 (3.8%) were current users.

#### Female Participants

Participants were team- (*n* = 60) or individual- (*n* = 81) sport athletes or hardcore- (*n* = 23) or corporate- (*n* = 75) gym exercisers, with ages ranging from 18 to 65 years (*M* = 26.82, *SD* = 11.35). They had been training/competing for an average of 8.54 years (*SD* = 6.38), spent an average of 7.67 h (*SD* = 4.34) per week training, and had trained in their current gym/with their current team for an average of 4.07 years (*SD* = 4.87). 223 (93.3%) participants reported never having used PED, 10 (4.2%) had used them prior to the past 3 months, three (1.3%) had used them in the past 3 months, and three (1.3%) were current users.

#### Individual-Sport Participants

Participants were male (*n* = 88) or female (*n* = 81) individual-sport athletes, with ages ranging from 18 to 63 years (*M* = 26.68, *SD* = 11.30). They had been training/competing for an average of 8.26 years (*SD* = 7.51), spent an average of 10.25 h (*SD* = 4.77) per week training, and had trained with their current club for an average of 4.41 years (*SD* = 5.00). 159 (94.1%) participants reported never having used PED, seven (4.1%) had used them prior to the past 3 months, one (0.6%) had used them in the past 3 months, and two (1.2%) were current users.

#### Team-Sport Participants

Participants were male (*n* = 135) or female (*n* = 60) team-sport athletes, with ages ranging from 17 to 41 years (*M* = 20.51, *SD* = 2.61). They had been training/competing for an average of 6.99 years (*SD* = 5.14), spent an average of 7.73 h (*SD* = 3.16) per week training, and had trained with their current team for an average of 3.03 years (*SD* = 3.05). 175 (89.7%) participants reported never having used PED, 13 (6.7%) had used them prior to the past 3 months, four (2.1%) had used in the past 3 months, and three (1.5%) were current users.

#### Hardcore-Gym Participants

Participants were male (*n* = 102) or female (*n* = 23) hardcore-gym exercisers, with ages ranging from 17 to 70 years (*M* = 27.97, *SD* = 9.17). They had been training/competing for an average of 6.90 years (*SD* = 6.16), spent an average of 9.89 h (*SD* = 4.52) per week training, and had trained in their current gym for an average of 4.19 years (*SD* = 4.53). 76 (60.8%) participants reported never having used PED, 23 (18.4%) had used them prior to the past 3 months, 15 (12.0%) had used them in the past 3 months, and 11 (8.8%) were current users.

#### Corporate-Gym Participants

Participants were male (*n* = 46) or female (*n* = 75) corporate-gym exercisers, with ages ranging from 18 to 73 years (*M* = 33.20, *SD* = 14.61). They had been training/competing for an average of 10.92 years (*SD* = 9.23), spent an average of 4.78 h (*SD* = 3.44) per week training, and had trained in their current gym for an average of 4.72 years (*SD* = 6.51). 117 (96.7%) participants reported never having used PED, three (2.5%) had used them prior to the past 3 months, none had used them in the past 3 months, and one (0.8%) was a current user.

### Measures

#### Doping MD

The doping MD scale – short (DMDS-S; Boardley et al., in review) was used to measure participants’ doping MD. This scale consists of six items (e.g., “Compared to most lifestyles in the general public, doping isn’t that bad”), with one item for each of the six MD mechanisms relevant to doping in sport/exercise. Participants were asked to read a number of statements describing thoughts and feelings that athletes may have and indicate their level of agreement with each statement using a Likert scale anchored by 1 (*strongly disagree*) and 7 (*strongly agree*). The scale has shown very good levels of internal consistency and test-retest reliability. Further, evidence for its factorial, convergent and discriminant validity has been provided (Boardley et al., in review).

#### Doping SRE

The doping SRE scale (DSRES; Boardley et al., in review) was used to assess doping SRE. This measure consists of six items (e.g., “Resist doping even if you knew you could get away with it?”) that assess peoples’ capacity to withstand personal and social influences encouraging the use of PED. For each item, participants rated their confidence in their ability to engage in relevant behaviors using a Likert scale anchored by 1 (*no confidence*) and 5 (*complete confidence*). The scale has shown very good levels of internal consistency and test–retest reliability. Further, evidence for its factorial, convergent and discriminant validity has been provided (Boardley et al., in review).

#### Guilt

To assess participants’ anticipated guilt responses to doping, participants were asked to imagine being in the following situation:

Having returned to training following a period of injury, you are feeling very out of shape. As such, you feel the need to get back in shape as soon as possible. A friend who you train with has been taking a training supplement that he/she says really helped him/her get back in shape quickly following a similar injury. He/she offers to give you some and you decide to take it. Subsequently you get back in shape much quicker than expected, but then discover the supplement you have been taking is a banned performance-enhancing substance. However, due to the improvements you have experienced, you decide to continue taking the substance.

Participants were then asked to indicate how they would anticipate feeling about continuing to take the substance by responding to the five items (e.g., “I would feel remorse, regret”) that form the guilt scale in the State Shame and Guilt Scale (SSGS; Marschall et al., 1994). Participants responded on a 5-point scale ranging from 1 (*not at all*) to 5 (*extremely*). Marschall et al. (1994) provided evidence supporting the construct validity and internal reliability of this sub-scale.

#### Empathy

Scores on the seven-item perspective taking (e.g., “Before criticizing somebody, I try to imagine how I would feel if I were in their place”) and seven-item empathic concern (e.g., “I am often quite touched by things that I see happen”) subscales of the Interpersonal Reactivity Index (Davis, 1983) were used to measure empathy. Participants were asked to indicate how well the statements described them and responded on a scale with anchors of 1 (*does not describe me well*) and 7 (*describes me very well*). This scale has been used in past research, and has been shown to be a valid and reliable measure of empathy (Carlo et al., 1999).

#### Reported Doping

Our approach to the assessment of reported doping was based on the method used by Lucidi et al. (2008). More specifically, participants were provided with a list of nine categories of doping substances (e.g., Ephedrine stimulants) and methods (e.g., Blood manipulation) and asked to indicate which ones they currently used, had used in the past 3 months, had used prior to the past 3 months or had never used. The list of doping substances was based on those banned in sport by the WADA. Participants’ responses were used to form a score from one to four, with participants being assigned a score of one if they indicated never using any of the substances/methods, two if they had used one or more of them but only prior to the past 3 months, three if they had used one or more of them in the past 3 months and four if they currently used one or more of the substances/methods.

#### Doping Susceptibility

Participants’ susceptibility to doping was assessed using the approach of Gucciardi et al. (2010). This approach involves presenting participants with the following scenario: “If you were offered a banned performance enhancing substance under medical supervision at low or no financial cost and the banned performance-enhancing substance could make a significant difference to your performance and was currently not detectable.” Participants are then asked to report how much consideration they would give to this offer on a scale from 1 (*none at all*) to 7 (*a lot of consideration*). Previous research has validated this method of assessing susceptibility to doping (Gucciardi et al., 2010).

### Procedures

Recruitment for the project commenced once ethical clearance was provided by the ethics committee of the lead author’s institution. Our approach to recruitment differed for sport versus exercise participants. For sport participants, we contacted team- and individual-sport coaches regarding participation of the athletes they coached. For coaches who agreed to allow access to their athletes we arranged a designated training session during which we introduced the project to athletes and invited them to participate. In contrast, exercise participants were recruited through managers of hardcore and corporate gymnasia who were asked whether it would be possible to invite exercisers at their gymnasia to participate in the study. Once access was agreed through gymnasia managers, potential participants were approached in the reception area of gymnasia and invited to participate. Before completing the questionnaire, all respondents were informed that the survey examined attitudes relating to sport/exercise and that honesty in responses was vital to the success of the study. It was also explained that all responses would be kept strictly confidential and would be used only for research purposes. Participants signed an informed consent form prior to completing the questionnaire, which took approximately 10–15 min to complete for each participant. All recruitment and data collection was conducted by one of three research associates.

## Results

### Data Screening, Descriptive Statistics, Scale Reliabilities, and Correlations

Preliminary data screening was conducted to check for missing values (Tabachnick and Fidell, 2001). 1.02% of the data were missing and missingness was unrelated to any variable, thus missing data were assumed to be missing at random. The expectation maximization algorithm was used to impute missing values prior to further data analysis.

Descriptive statistics, scale reliabilities and Pearson correlations for all study variables are presented in **Table 1**. On average, participants reported low-to-moderate levels of doping MD, high levels of doping SRE, moderate levels of empathy and anticipated guilt, low levels of reported doping and low-to-moderate levels of doping susceptibility. Next, the four psychometric questionnaires demonstrated good to excellent levels of internal consistency. Then, skewness and kurtosis values indicated most variables were normally distributed (Curran et al., 1996). This was not the case for the reported doping data though, which – as may be expected – demonstrated positive skew and kurtosis values due to a large proportion of the sample reporting never having used PED. Finally, significant Pearson correlations were observed between all study variables. Importantly, doping MD had strong, moderate, and very strong negative correlations, respectively, with doping SRE, empathy and anticipated guilt, and moderate and strong positive relationships, respectively, with reported doping and doping susceptibility. Further, empathy showed a moderate-to-strong positive association with anticipated guilt. Finally, anticipated guilt had moderate-to-strong and strong negative associations, respectively, with reported doping and doping susceptibility.

**Table 1:** Descriptive statistics, scale reliabilities, and correlations (*N* = 610).

Variable	*M*	*SD*	Range	Skew	Kurtosis	1	2	3	4	5
(1) Doping moral disengagement	2.52	1.22	1.00–7.00	1.14	1.30	(0.88)				
(2) Doping self-regulatory efficacy	4.53	0.72	1.00–5.00	-1.88	3.30	-0.45	(0.93)			
(3) Empathy	4.60	0.75	1.43–6.57	-0.25	0.63	-0.27	0.24	(0.79)		
(4) Anticipated guilt	3.85	1.14	1.00–5.00	-1.01	0.11	-0.62	0.43	0.39	(0.95)	
(5) Reported doping	1.22	0.64	1.00–4.00	3.11	9.22	0.33	-0.20	-0.17	-0.37	–
(6) Doping susceptibility	2.83	1.97	1.00–7.00	0.80	-0.63	0.54	-0.41	-0.24	-0.54	0.32

*For all correlations, *p* < 0.01*.

### Model Testing

To test the hypothesized model, structural equation modeling (SEM) was employed. All Structural Equation Modeling (SEM) analyses were conducted using the EQS 6.1 statistical package with the maximum likelihood estimator (Bentler and Wu, 2002). Indices used to estimate model fit for each model were the Satorra–Bentler scaled robust chi-square (Rχ^2^), the robust comparative fit index (RCFI), the standardized root mean square residual (SRMR), and the root mean square error of approximation (RMSEA). Good model fit is attained when RCFI values are close to or above 0.95, the RMSEA is less than 0.06, and the SRMR is less than 0.08 (Hu and Bentler, 1999). To compare models, the robust consistent Akaike information criterion (RCAIC) was used. When making comparisons between nested models, the model with the lowest value is preferred (Hair et al., 1998). When conducting the SEM analyses, the two-step approach recommended by Anderson and Gerbing (1988) was employed. The first step involves testing the measurement model, which includes the postulated relationships of the observed variables to their respective latent constructs and allows all latent constructs to intercorrelate. In initial analyses the normalized estimate of Mardia’s coefficient indicated substantial deviation from multivariate normality. Thus, the Robust Maximum Likelihood estimation method was used for all analyses, as this method provides more accurate standard errors, chi-squared values, and fit indices when data are non-normally distributed (Bentler and Wu, 2002). The case numbers with the largest contribution to normalized multivariate kurtosis suggested minimal impact of outliers and as a result no cases were deleted. The measurement model specified included the six items of the DMDS-S, the six items of the DSRES, the best six indicators of empathy (three items for empathic concern and three for perspective taking; determined through factor loadings and modification indices during CFA [see Hofmann, 1995]), the five items from the SSGS and one item for reported doping. Specification of this model resulted in an excellent model fit, χ^2^(241) = 401.09, *p* < 0.05; CFI = 0.971; RMSEA = 0.033; SRMR = 0.041.

We then proceeded to the second step in Anderson and Gerbing’s approach, which involves testing a model incorporating the hypothesized structural pathways. Specification of the structural model resulted in an excellent model fit, χ^2^(244) = 407.35, *p* < 0.05; CFI = 0.970; RMSEA = 0.033; SRMR = 0.044. As shown by the standardized coefficients (see **Figure 1**), empathy and doping SRE, respectively, had weak-to-moderate and moderate-to-strong negative predictive effects on doping MD, empathy and doping MD, respectively, had weak-to-moderate positive and strong-to-very strong negative predictive effects on anticipated guilt, and doping MD and anticipated guilt, respectively, had weak-to-moderate positive and moderate negative predictive effects on doping use. Overall the model accounted for 31% of the variance in doping MD, 51% of the variance in anticipated guilt and 18% of the variance in doping use.

### Mediational Analyses

To investigate the extent to which predictive effects operated via the mediational paths shown in **Figure 1**, when specifying each model in EQS we requested the decomposition of model effects into direct, indirect, and total effects (Bollen, 1987); the statistical significance of these effects was determined as part of model testing in EQS. For the effect of empathy on anticipated guilt via doping MD, the total, direct, and indirect effects were 0.35 (*p* < 0.05), 0.23 (*p* < 0.05), and 0.12 (*p* < 0.05), respectively; the percentage of the total effect mediated by doping MD was 34%. Next, for the effect of doping SRE on anticipated guilt via doping MD, the total, direct, and indirect effects were 0.27 (*p* < 0.05), 0.00 (*p* > 0.05), and 0.27 (*p* < 0.05), respectively; the percentage of the total effect mediated by doping MD was 100%. Then, for the effect of doping MD on doping use via anticipated guilt, the total, direct, and indirect effects were 0.36 (*p* < 0.05), 0.20 (*p* < 0.05), and 0.16 (*p* < 0.05), respectively; the percentage of the total effect mediated by anticipated guilt was 44%. Next, for the effect of doping SRE on doping use via doping MD and anticipated guilt, the total, direct, and indirect effects were -0.16 (*p* < 0.05), 0.00 (*p* > 0.05), and -0.16 (*p* < 0.05), respectively; the percentage of the total effect mediated by anticipated guilt was 100%. Finally, for the effect of empathy on doping use via doping MD and anticipated guilt, the total, direct, and indirect effects were -0.13 (*p* < 0.05), 0.00 (*p* > 0.05), and -0.13 (*p* < 0.05), respectively; the percentage of the total effect mediated by anticipated guilt was 100%.

### Multigroup Analyses

When testing structural models in diverse populations, it is important to determine the equivalence of the final model across different subgroups within the overall population (Cheung and Rensvold, 2002). As such, in the current analyses we tested for measurement and structural invariance of the final model between males and females and across four sport and exercise sub-groups. Although we were primarily interested in structural invariance, in order to test structural invariance, it is important to first determine the measurement invariance of the psychometric measures employed. As such, we tested four relevant aspects of invariance (Cheung and Rensvold, 2002; Byrne, 2006): (a) configural invariance, which exists when the items of a scale are indicators of the same factors in different groups; (b) metric invariance, which is present when all factor loadings are equal across groups; (c) equivalence of construct variance and covariance across the two genders, which determines whether the variances and covariances of the latent variables are equivalent across groups; and (d) *structural invariance*, which determines whether model fit is affected when all structural components in the model are constrained to be equal across groups. To compare fit between more- and less-constrained models we used ΔCFI, with values of less than -0.01 indicating no significant difference between models (Cheung and Rensvold, 2002). Due to the low levels of variance in reported doping in some of the sub-samples (see method section) in these analyses we used doping susceptibility as our outcome variable in place of reported doping. The results of these analyses are shown in **Table 2**.

**Table 2:** Fit indices for multisample analyses on the structural process model.

Model	*df*	Rχ^2^	Rχ^2^/*df*	RCFI	SRMR	RMSEA	RCAIC
**Gender**					
Baseline males	244	368.52	1.51	0.968	0.045	0.037	-1319.04
Baseline females	244	296.23	1.21	0.973	0.060	0.030	-1284.02
Configural invariance	488	665.44	1.36	0.969	0.053	0.035	-2764.88
Metric invariance	507	694.22	1.37	0.967	0.059	0.035	-2865.58
ECVC	510	731.18	1.43	0.962	0.148	0.038	-3049.68
ECVC revised	508	696.54	1.37	0.967	0.061	0.035	-3069.49
Structural invariance	514	716.49	1.39	0.965	0.082	0.036	-3094.02
**Sport/exercise group**					
Baseline corporate	244	317.00	1.30	0.917	0.070	0.050	-1097.17
Baseline hardcore	244	316.49	1.30	0.969	0.052	0.049	-1105.62
Baseline team	244	332.79	1.36	0.941	0.070	0.043	-1197.82
Baseline individual	244	345.29	1.42	0.913	0.074	0.050	-1150.40
Configural invariance	976	1312.40	1.34	0.939	0.067	0.048	-5923.14
Metric invariance	1033	1407.41	1.36	0.932	0.087	0.049	-6250.70
ECVC	1042	1437.85	1.38	0.928	0.136	0.050	-6286.98
ECVC revised	1039	1426.41	1.37	0.929	0.112	0.050	-6276.17
Structural invariance	1057	1469.00	1.39	0.925	0.143	0.051	-6367.02
Structural invariance revised	1054	1443.30	1.37	0.929	0.112	0.049	-6370.49

*Rχ^*2*^, Satorra–Bentler scaled chi-square; RCFI, robust comparative fit index; SRMR, standardized root mean square residual; RMSEA, root mean square error of approximation; RCAIC, robust consistent Akaike information criterion; ECVC, equivalence of construct variance and covariance. For all models, Rχ^*2*^*p* < 0.05*.

#### Gender Invariance

Model fit for the baseline models was very good for both males and females, and configural invariance was demonstrated by the very good fit of the relevant model. Next, metric invariance was also established by the very good fit of this model. However, specifying the equivalence of construct variance and covariance resulted in a significant reduction in model fit. Inspection of the modification indices identified constraining the variance of doping SRE and the covariance between doping SRE and empathy to equivalence had both contributed to the reduction in fit. Releasing these constraints in a revised model resulted in very good model fit (Van de Schoot et al., 2012). Finally, subsequent constraining of the structural components of the model to equivalence between males and females resulted in a model with very good model fit, demonstrating structural invariance between the two genders.

#### Sport/Exercise Group Invariance

Model fit for the baseline models ranged from acceptable-to-good for corporate-gym exercisers to very good for hardcore-gym attendees. Then, configural invariance was established by the good model fit for this model. Next, metric invariance was also established, as illustrated by the good fit of this model. However, the equivalence of construct variance and covariance was not established, as specifying these constraints across the four groups resulted in a ΔCFI > 0.01. Inspection of the modification indices identified constraining the variance of doping SRE to equivalence across the four groups had caused the reduction in fit. Releasing these constraints in a revised model resulted in acceptable model fit. Then, constraining the structural components of the model to equivalence across the four groups also resulted in a ΔCFI > 0.01. Inspection of the modification indices indicated the path from empathy to doping MD was variant between hardcore-gym exercisers and the other three groups. Specification of a model with these constraints released resulted in a model with acceptable model fit and no further variant constraints indicated. This model also indicated the nature of the divergent path coefficients, with the standardized coefficient for the path from empathy to doping MD being stronger in hardcore-gym exercisers (i.e., -0.45, *p* < 0.05) than corporate-gym exercisers (i.e., -0.27, *p* < 0.05), team-sport athletes (i.e., -0.11, *p* > 0.05) and individual-sport athletes (i.e., 0.08, *p* > 0.05).

## Discussion

Both qualitative and quantitative research has highlighted the potential importance of doping MD to the regulation of doping in sport and exercise contexts (e.g., Lucidi et al., 2004, 2008; Zelli et al., 2010; Hodge et al., 2013; Boardley and Grix, 2014; Boardley et al., 2014, 2015). However, to date, researchers have not investigated the role of doping MD alongside other variables from Bandura’s (1991) theory (e.g., empathy, guilt) when investigating correlates of doping, nor have they investigated these variables across a range of sport and exercise contexts. The present research sought to address these deficits in knowledge by testing a model of doping behavior based on Bandura’s (1991) theory with a sample of team- and individual-sport athletes and corporate- and hardcore-gym exercisers. We also sought to examine whether the model was invariant between males and females and across the four sport/exercise groups represented. Over the coming paragraphs, we review and discuss the key findings from the research pertaining to these aims.

The primary aim of the current research was to test a model of doping behavior with team- and individual-sport athletes and corporate- and hardcore-gym exercisers. Grounded in Bandura’s (1991) theory, the hypothesized process model (see **Figure 1**) depicted empathy and SRE would negatively predict doping MD, doping MD would negatively predict anticipated guilt, and anticipated guilt would negatively predict reported doping (Bandura, 1991; Bandura et al., 1996, 2001; Lucidi et al., 2008; Zelli et al., 2010; Stanger et al., 2012; Paciello et al., 2013). In addition, doping SRE and empathy would negatively predict doping indirectly via changes in doping MD and anticipated guilt. Further, empathy was proposed to have a direct positive effect on anticipated guilt. Finally, doping MD was projected to positively predict doping directly and indirectly through anticipated guilt (Bandura, 1991; Bandura et al., 1996). Overall, data analyses supported the efficacy of this model, and the meaning and implications of the relevant findings are subsequently discussed.

One of the major contributions of this study was the strong support it provided for the main tenets of Bandura’s (1991) theory. Although researchers have tested some of the more holistic aspects of this theory in other contexts (e.g., Bandura et al., 1996, 2001), doping researchers have instead investigated the predictive effects of MD (e.g., Hodge et al., 2013) and SRE (Lucidi et al., 2004, 2008) on doping-related outcomes within process models based primarily on other theories. By investigating the predictive effects of these variables in a model that included further key aspects of Bandura’s (1991) theory, we were able to determine whether the processes through which doping MD may operate are consistent with this theory. The support we found for our hypothesized model provides evidence of the relevance of other aspects of Bandura’s (1991) theory for doping in sport and exercise, namely empathy and anticipated guilt. Another major strength of the current work was that we purposefully tested our model in a sample that represented a range of relevant populations, including ones in which doping is highly prevalent. This too contrasts with past work investigating doping MD and doping SRE, which has either sampled from populations in which the prevalence of doping was extremely low (e.g., Lucidi et al., 2004, 2008) or the prevalence of doping was not assessed (Hodge et al., 2013). Thus, the current work demonstrates the relevance of Bandura’s (1991) theory in athletic populations in which there is a demonstrable need to understand the psychosocial factors that facilitate doping.

Regarding the specific predictive effects shown in model testing, support was found for empathy being a possible antecedent of both doping MD and anticipated guilt. More specifically, weak-to-moderate predictive effects of empathy on these variables were demonstrated. The predictive effect of empathy on doping MD was negative, supporting Bandura’s (1986, 1991) contention that higher levels of empathy lead to lower levels of MD and less frequent transgressive behavior. Endorsement of and engagement in deleterious conduct is more difficult when one can anticipate and experience the consequences of one’s actions for others. This is the first study to show this effect in the specific context of doping, and in sport or exercise more generally. However, it is consistent with empirical work investigating unethical business decisions and youth antisocial behavior (Detert et al., 2008; Hyde et al., 2010). Using a sample of business students, Detert et al. (2008) showed empathy to be a negative predictor of MD. Similarly, Hyde et al. (2010) employed a prospective design to show empathy at age 12 negatively predicted MD at age 15 in youth from low-income families. In contrast to those on doping MD, the predictive effects of empathy on anticipated guilt were positive. This is a further novel finding, as researchers to date have not investigated empathy as a precursor of anticipated guilt in doping research. These variables have been empirically linked in children though, with more empathic children showing higher levels of guilt (Roberts et al., 2014). Thus, the associations between empathy, MD and guilt in the current study are consistent with theory and empirical work in other contexts, and suggest empathy may be important to our understanding of doping behavior.

The possible role of doping SRE as an antecedent of doping MD was also supported during model testing, as doping SRE had a strong negative predictive effect on doping MD. Thus, participants who had stronger beliefs in their capacity to withstand personal and social influences encouraging doping reported lower levels of doping MD. This effect is consistent with the assertion made by Bandura et al. (2001) that increased SRE leads to lower levels of MD. According to Bandura et al. (2001), those who are confident in their ability to resist incentives to transgress have no need to develop the skills required to cognitively restructure detrimental conduct through MD. Further, Bandura et al. (2001) also demonstrated a weak negative effect of peer pressure SRE on MD in the context of delinquent behavior in children. Our finding extends this predictive effect to the context of doping across a range of key sport and exercise populations.

In terms of the predictive abilities of doping MD, this variable was found to predict both anticipated guilt and reported doping. Regarding anticipated guilt, doping MD had a very strong negative predictive effect on this variable. This shows that participants who had a greater tendency to agree with justifications and rationalizations for doping were less likely to anticipate experiencing guilt for choosing to dope. This finding provides strong support for this key aspect of Bandura’s (1991) theory, and provides the first empirical evidence of its relevance to doping. It is, however, consistent with research in other contexts (e.g., children’s interpersonal aggression and delinquent conduct), which has shown a negative predictive effect of MD on guilt (Bandura et al., 1996). In addition to its effect on anticipated guilt, doping MD also had a weak-to-moderate positive effect on reported doping, such that participants with higher levels of doping MD were more likely to report having taken PED. This finding provides statistical evidence to support qualitative research that links MD with PED use in male bodybuilders and team- and individual-sport athletes (Boardley and Grix, 2014; Boardley et al., 2014, 2015). It is also consistent with research on children’s interpersonal aggression and delinquent conduct, which also found MD not only predicted guilt, but also had a direct positive predictive effect on delinquent behavior (Bandura et al., 1996). Similarly, in both the current research and Bandura et al. (1996), anticipated guilt was found to have a negative predictive effect on the target behavior. These findings support another aspect of Bandura’s (1991) theory, which suggests increased levels of anticipated guilt should deter transgressive and harmful conduct. As such, collectively these findings provide further support for the potential relevance of Bandura’s (1991) theory to our understanding of the psychosocial factors that govern doping behavior.

In addition to the direct effects already discussed, there were also a number of indirect (i.e., mediated) associations identified during model testing. First, empathy had a weak positive predictive effect on anticipated guilt via doping MD, such that when participants had higher levels of empathy, associated increases in anticipated guilt were explained through lower levels of doping MD. This mediated effect has been identified in past research in other contexts. Specifically, Detert et al. (2008) showed reduced MD mediated a negative effect of empathy on unethical decision making in business students. Also, Hyde et al. (2010) employed a prospective design to show MD at age 15 meditated an effect of empathy at age 12 on youth antisocial behavior at age 16–17. Integrating this finding with Bandura’s (1991) theory, this suggests when athletes and exercisers are more adept at understanding and experiencing the implications of their actions for others, they may be less able to rationalize and justify doping, which in turn may lead to increased anticipation of guilt for doping. Similarly, doping SRE had a moderate positive predictive effect on anticipated guilt via doping MD, meaning the tendency for participants with higher levels doping SRE to have higher levels of anticipated guilt could be explained through lower levels of doping MD. Although this specific indirect effect has not been tested previously, research has negatively linked peer-pressure SRE with MD (see Bandura et al., 2001), and MD has been positively associated with transgressive behavior (Bandura et al., 1996). Interpreting this finding in the current work suggests athletes and exercisers who are more able to resist internal and external pressures to dope should anticipate feeling more guilt for doping because of their reduced tendency to rationalize and justify doping.

The third indirect effect was a weak positive effect of doping MD on reported doping via anticipated guilt. As such, the increased levels of reported doping in participants with higher levels of doping MD could be explained through lower levels of anticipated guilt. Consistent with this effect, Bandura et al. (2001) found guilt to mediate the effect of MD on children’s delinquent behavior. Presently, such a pathway suggests athletes and exercisers who have higher levels of doping MD may be more likely to dope because justifying and rationalizing doping allows them to do so without anticipating deterrent emotions such as guilt. Finally, both empathy and doping SRE had weak negative predictive effects on reported doping via their relationships with doping MD and anticipated guilt. As such, both of these variables may influence doping though the combined effects of some of the previously discussed indirect effects. In sum, it is important to consider both the direct and indirect effects operating in **Figure 1**, and to keep in mind all variables in the hypothesized model had predictive effects on reported doping either directly and/or via their links with other variables.

Although the multisample analyses largely supported the invariance of the structural model across gender and sport/exercise type, they did present evidence of one divergent path in the sport/exercise-type analyses. More specifically, there were apparent differences in the strength of the path from empathy to doping MD across the four sport/exercise groups; this path was strong and negative for hardcore-gym exercisers, moderate and negative for corporate-gym exercisers and non-significant for both team- and individual-sport athletes. Interestingly, past research has shown differences in levels of empathy between steroid users and non-users that could be relevant to these group differences. Moreover, steroid users have been found to be lower in empathy than non-users (Porcerelli and Sandler, 1995). Given doping was more prevalent in the hardcore-gym exercisers than in the other three groups, it is possible this group as a whole had lower levels of empathy. As such, it may be that in groups in which empathy is generally lower, any increases in this variable may have a more powerful deteriorating effect on doping MD, in comparison to groups where levels of empathy are generally higher. Given empathy has been shown to be trainable in groups with low levels of empathy (Hepper et al., 2014), future researchers are encouraged to specifically test the causal nature of this path, and also identify particular groups in which empathy-based interventions may potentially be most effective at reducing doping MD.

### Limitations and Future Research Directions

In accomplishing its aims, this research revealed a number of interesting and important findings. As with any research, however, these results should be considered alongside certain limitations resulting from the research design. First, given that the model-testing aspects of the project were based on cross-sectional data, the causal nature of the predictive effects identified could not be tested and therefore should not be inferred. With this in mind, future researchers are encouraged to build upon our work by employing experimental or quasi-experimental designs to test the causal nature of the identified associations. Longitudinal research testing the temporal ordering proposed in the model tested would also be a worthwhile direction for future work. Next, our use of self-report measures means the precision of the reported associations are in part reliant on participants’ honesty and introspective abilities to provide accurate responses to questionnaire items. This is especially an issue for the assessment of doping behavior, which given its socially sensitive nature can be particularly susceptible to under-reporting when assessed through self-report (de Hon et al., 2015). However, no method of assessing doping behavior is without limitation (see de Hon et al., 2015 for a discussion). What is important is to be aware of the relevant limitations when interpreting research on doping behavior, and that researchers use a range of approaches to its assessment. As such, researchers are encouraged to further test our findings by employing alternative approaches, such as physiological testing for evidence of doping or assessment of automatic associations as indicators of implicit responses to doping (see Petróczi, 2013). Future researchers should also assess social desirability so any effects of this construct can be accounted for during statistical testing. A further limitation relates to our use of a single-item measure of doping susceptibility for our multisample analyses. Although the validity of this measure has been supported in past research (Gucciardi et al., 2010), it is not possible to determine the internal reliability of single-item measures such as this. Future researchers should therefore explore alternative approaches to the assessment of doping susceptibility.

## Conclusion

By being the first study to examine the predictive effects of doping SRE and doping MD on doping use and susceptibility to doping alongside other key elements of Bandura’s (1991) theory, the current research has made a significant contribution to our understanding of the psychosocial factors associated with PED use. Importantly, these key elements – empathy and anticipated guilt – were previously untested with regard to their possible roles in governing doping behavior. The strong support we found for our hypothesized process model demonstrates the potential utility of Bandura’s (1991) theory in aiding our understanding of the psychosocial processes that facilitate doping in sport and exercise. Future researchers are therefore encouraged to build on our findings by testing the identified effects using experimental and longitudinal designs, and by employing alternative approaches to assess doping behavior.

## Ethics Statement

This study was carried out in accordance with the ethical guidelines and recommendations of The British Psychological Society with written informed consent from all subjects. All subjects gave written informed consent in accordance with the Declaration of Helsinki. The protocol was approved by the science, technology, engineering and mathematics ethical review committee of the University of Birmingham, United Kingdom.

## Author Contributions

IB led the development and implementation of the project. IB, AS, and JG collaborated to develop and design the project. CW collected the data for the project. IB conducted the data analysis, in collaboration with AS. IB wrote the initial draft of the paper, and all other authors provided comments/edits on the initial draft and therefore contributed to the final manuscript.

## Conflict of Interest Statement

The authors declare that the research was conducted in the absence of any commercial or financial relationships that could be construed as a potential conflict of interest.

## Footnotes

1 This estimated range would likely be lower for self-reported data, such as those reported presently (de Hon et al., 2015).

2 Doping behavior is defined presently as self-reported use of one or more of the prohibited substances/methods specified by the WADA.

3 The two remaining moral disengagement mechanisms are dehumanization and attribution of blame.

4 Some of the data from the current paper were also used to develop and validate the measures of doping SRE and doping MD employed presently (Boardley et al., in review). However, not all variables reported presently were used in the development and validation process, and none of the analyses reported presently were used to develop and validate the measures.
